# Chimeric Antigen Receptor (CAR)-Modified Immune Effector Cell Therapy for Acute Myeloid Leukemia (AML)

**DOI:** 10.3390/cancers12123617

**Published:** 2020-12-03

**Authors:** Utkarsh H. Acharya, Roland B. Walter

**Affiliations:** 1 Divisions of Hematologic Malignancies & Immune Effector Cell Therapy, Department of Medical Oncology, Dana-Farber Cancer Institute, Boston, MA 02215, USA; 2 Department of Medicine, Harvard Medical School, Boston, MA 02215, USA; 3 Clinical Research Division, Fred Hutchinson Cancer Research Center, Seattle, WA 98109, USA; rwalter@fredhutch.org; 4 Department of Medicine, Division of Hematology, University of Washington, Seattle, WA 98195, USA; 5 Department of Laboratory Medicine & Pathology, University of Washington, Seattle, WA 98195, USA; 6 Department of Epidemiology, University of Washington, Seattle, WA 98195, USA

**Keywords:** acute myeloid leukemia, adoptive cell transfer, antigen, antitumor efficacy, chimeric antigen receptor (CAR), microenvironment, natural killer cell, resistance, safety, T cell

## Abstract

**Simple Summary:**

Adoptive cell transfer with chimeric antigen receptor (CAR)-modified immune effector cells (IECs) has quickly emerged as a paradigm-shifting approach for the management of B cell malignancies given its ability to induce high rates of remission. This is reflected by the regulatory approval of three CD19-directed CAR T cell products to date for the treatment of several non-Hodgkin lymphomas and pediatric/young adult B-acute lymphoblastic leukemia (B-ALL). While fueled by this success, the use of CAR-modified IECs in acute myeloid leukemia (AML) is still in its infancy, with recognized challenges involving the selection of suitable target antigens, immune resistance due to a hostile tumor microenvironment, and potentially fatal toxicity to normal cells, in particular hematopoietic cells. Here, we will summarize the emerging landscape and therapeutic challenges with the use of such cells in patients with AML.

**Abstract:**

Despite the availability of an increasing number of targeted therapeutics and wider use of allogeneic hematopoietic stem cell transplantation, many patients with acute myeloid leukemia (AML) ultimately succumb to this disease. Given their remarkable efficacy in B-acute lymphoblastic leukemia and other CD19-expressing B cell malignancies, there is hope adoptive cellular transfer, particularly chimeric antigen receptor (CAR)-modified immune effector cell (IEC) therapies, may afford a novel, potent immune-based approach for the treatment of AML that complements or replaces existing ones and improves cure rates. However, it is unclear how best to translate the success of these therapies from B cell malignancies, where use of highly potent immunotherapies is facilitated by identified target antigens with near ubiquitous expression on malignant cells and non-fatal consequences from “on-target, off-tumor cell” toxicities. Herein, we review the current status of CAR-modified IEC therapies for AML, with considerations regarding suitable, relatively leukemia-restricted target antigens, expected toxicities, and interactions of the engineered cells with a profoundly immunosuppressive tumor microenvironment that restricts their therapeutic efficacy. With these challenges in mind, we will discuss possible strategies to improve the cells’ potency as well as their therapeutic window for optimal clinical use in AML.

## 1. Introduction

The principles of modern curative-intent therapy for acute myeloid leukemia (AML) were established in the early 1970s with the introduction of intensive combination chemotherapy with cytarabine and an anthracycline (“7 + 3”) [1]. With advancements in supportive care and refinements in the delivery of hematopoietic cell transplantation (HCT), treatment outcomes have gradually improved over the past 5 decades, particularly for children and younger adults [2,3,4,5,6]. Invariant for many years, the treatment landscape is currently rapidly changing. Largely, this is because the U.S. Food & Drug Administration (FDA) has approved nine new drugs for AML in the last 3 years: CC-486, CPX-351, enasidenib, gemtuzumab ozogamicin, gilteritinib, glasdegib, ivosidenib, midostaurin, and venetoclax [7,8,9,10,11,12,13,14,15,16]. At least some of these drugs are available in many other countries as well. It is expected survival will further increase with integration of these therapeutics into the evolving treatment schemes. However, despite the widespread availability and use of these agents, AML remains difficult to cure, and deaths from persistent or recurrent disease remain frequent. Thus, the need for new, effective therapeutic strategies that complement or replace existing ones and improve outcomes, including cure rates, remains undisputed.

Of immense current interest to accomplish this goal is adoptive cell transfer, i.e., the infusion of immune cells with direct antitumor activity, in particular those involving chimeric antigen receptor (CAR)-modified immune effector cells (IECs). This interest is partly founded on the well-established curative potential of allogeneic HCT via immune cell-mediated cytotoxicity (“graft-versus-leukemia” effect). Much of the enthusiasm for exploiting CAR-modified IECs in AML, however, comes from their success in the management of various B cell malignancies where CD19-directed CAR T cell and, most recently, natural killer (NK) cell products have shown exquisite efficacy without the risk and associated morbidity and mortality of graft-versus-host disease (GVHD) intrinsic to allogeneic HCT. Unprecedented responses and remission rates seen in B-acute lymphoblastic leukemia (B-ALL) and several mature B cell lymphomas with CAR-modified T cell therapies led to the approvals of tisagenlecleucel, axicabtagene ciloleucel, and brexucabtagene autoleucel by the FDA and other regulatory authorities [17,18,19] and integration of such therapies into clinical practice.

How best to translate the success of adoptive cell transfer from B cell malignancies to AML is a matter of intense investigation. Targeting lymphoid tumors with CD19-directed CAR-modified IECs is effective and clinically feasible not only because of the near ubiquitous cell surface expression of CD19 on malignant B cells, but also because of the nonfatal consequences of “on-target, off-tumor cell” toxicity imposed by these engineered cell products towards normal B cells (B cell aplasia) with available supportive care measures (e.g., immunoglobulin substitution for hypogammaglobulinemia). A similarly ideal target antigen has not yet been identified in AML. With currently exploited antigens, adoptive cell therapies for AML are complicated by the overlapping antigen expression between malignant and normal myeloid cells, with resulting infection risks due to prolonged myelosuppression or myeloablation. Furthermore, the hostile tumor microenvironment comprised of metabolic limitations and abrogative cellular disruptions against IECs adds another potential challenge in the realm of AML. Herein, we review the current status of adoptive cell therapies for AML, with considerations regarding suitable target antigens, therapeutically relevant interactions of the engineered cells with the AML microenvironment, and possible strategies to improve the cells’ therapeutic window and efficacy when clinically used, focusing on CAR T cell therapies.

## 2. Brief Overview of Adoptive Cell Transfer with CAR-Modified IECs

Interest in adoptive cell transfer as a modality of cancer therapy started with the demonstration that ex vivo expanded populations of tumor-infiltrating lymphocytes have antitumor efficacy and can eradicate metastatic tumors [20,21,22,23,24,25]. Considering that immunologic graft-versus-leukemia effects imparted by allogeneic IECs have long been an important part of curative-intent therapy of myeloid neoplasms, it is not surprising there is great interest in developing effective and safe antigen-specific adoptive cell transfer approaches for patients with AML. Advances in gene transfer technologies made over the last two decades now enable the efficient redirection of IECs toward defined cancer antigens to enhance their antitumor efficacy and overcome the consequences of immune tolerance seen with the transfer of unmanipulated cells. For patients with hematologic malignancies, attention has focused on the utilization of CAR constructs expressed in IECs, primarily T cells, although there is increasing interest in using CAR constructs in engineered NK cells as well [26,27]. An alternative approach involves the use of T cells carrying a genetically engineered tumor-recognizing T cell receptor (TCR) [20,21,22,23,24,25]. As of 3 November 2020, there were close to 30 open or near-open CAR T cell trials listed in clinicaltrials.gov, highlighting the interest with antigen-specific engineered IECs in AML.

### 2.1. Adoptive Cell Transfer of TCR- and CAR-Engineered T Cells

**Figure 1:**
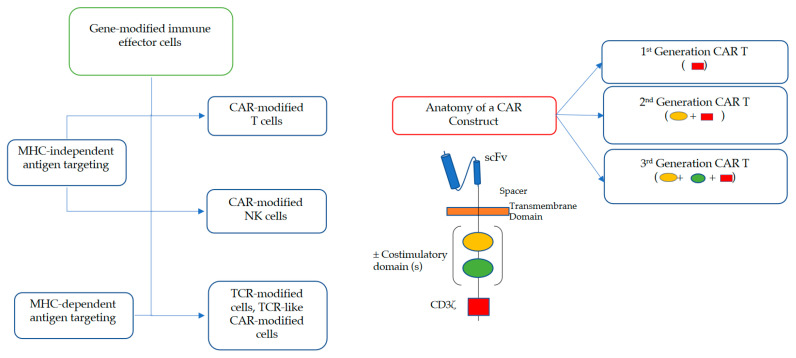
Types of gene-modified immune effector cell (IEC) constructs exerting cytotoxicity in a major histocompatibility complex (MHC)-independent or MHC-dependent manner as well as anatomy of chimeric antigen receptor (CAR) constructs.

Two main engineering strategies are currently pursued to render T cells more potent against AML (Figure 1). In the first, a genetically engineered TCR is used that recognizes a tumor-associated antigen [28,29,30]. Unlike CAR constructs, engineered TCRs are not modified at the intracellular domain. Therefore, TCR-modified cells, which can target human leukocyte antigen (HLA)-presented peptide epitopes from both intracellular as well as extracellular antigens, retain natural signaling properties. While TCR-modified T cells have been primarily exploited in patients with various solid tumors (e.g., synovial cell carcinoma and melanoma) [29,30], some data from the limited studies done to date in patients with AML, e.g., from a small trial testing an HLA A*02:01-restricted high-affinity Wilms’ Tumor Antigen-1 (WT1)-specific TCR expressed in Epstein–Barr virus (EBV)-specific donor CD8+ T cells in AML patients following allogeneic HCT [31], suggest potential usefulness in this malignancy as well. Clinical efforts with TCR-engineered cells directed at other antigens (e.g., PRAME (preferentially expressed antigen in melanoma) or HA-1) are ongoing (e.g., NCT02203903, NCT02494167, and NCT02743611), as are preclinical studies involving a number of additional antigens [28].

Currently more widely explored than TCR engineering is a second engineering strategy to increase the efficacy of T cells against AML, namely one that includes the use of CAR constructs (Figure 1). Fundamentally, CAR constructs are hybrid single-chain receptor constructs consisting of an ectodomain which recognizes a tumor antigen via antibody-derived singe-chain variable fragment (scFv), a transmembrane domain which houses a hinge molecule, and an endodomain that facilitates IEC signaling with or without costimulatory molecules. First-generation CAR constructs comprise of an antigen-binding ectodomain linked to an intracellular signaling domain (commonly the invariant CD3ζ chain of the T cell receptor). Second-generation CAR constructs, such as those used in the FDA-approved products, consist of an additional costimulatory molecule fused to CD3ζ such as CD27, CD28, CD134 (OX40), or CD137 (4-1BB). The latter influence in vivo expansion and persistence of the genetically modified T cells. Third-generation CAR constructs include multiple costimulatory domains and are under development [25,32,33,34,35].

In contrast to TCR-based therapies, CAR T cells are capable of exerting cytotoxicity in an HLA-unrestricted manner which theoretically allows for broader patient selection. To date, various iterations of CAR T cell delivery are being investigated across several diseases and disease stages with nuances in the presence, type, and number of costimulatory domains; T cell donor source (i.e., allogeneic vs. autologous); method of cellular transduction (i.e., viral, nonviral, transient, stable); cell dosing; and preparative lymphodepleting chemotherapy.

Clinically approved autologous CAR T cells have yielded exceptional responses in patients with B cell malignancies with potential for durable responses in the setting of long-term cellular persistence. In contrast to the use of allogeneic cells, autologous CAR T cells enable a potent immune-based cellular therapy approach without the unwanted risks of GVHD. However, their utility in highly proliferative diseases such as AML imposes a logistical challenge in the delivery of these therapies given the prolonged manufacturing time needed for cell engineering and expansion. Thus, allogeneic CAR T cells are being heavily researched with gene editing strategies that remove αβ TCRs on T cells from third party donors to attenuate GVHD risks and allow for readily available “off-the-shelf” therapies with minimal preemptive logistical planning.

### 2.2. Adoptive Cell Transfer of Engineered NK Cells

NK cells are cytotoxic lymphocytes of the innate immune system able to eliminate virally infected as well as oncogenically transformed cells and enhance antibody and T cell responses [36,37]. NK cells can be derived from a number of autologous and allogeneic cell sources, including peripheral blood, umbilical cord blood or postpartum placenta, CD34+ hematopoietic progenitor cells, human-induced pluripotent stem cells, and cell lines (e.g., NK-92 cells) for use in adoptive cell therapies [36,37,38]. There is growing interest in allogeneic NK cells after it was realized that autologous cells expand in vivo but typically show insufficient antitumor efficacy. A particular appeal in their clinical use is that NK cells are highly cytotoxic effector cells yet do not cause GVHD [39] and, therefore, do not require HLA-matching. While currently not nearly as widely pursued as CAR T cells for adoptive cell transfer with engineered IECs, there are increasing efforts in fortifying NK cells with CAR constructs to enhance their potency against malignant cells [36,37]. Again, CD19 has served as paradigm target antigen for these efforts, with an increasing number of studies demonstrating significant antitumor efficacy of CAR-modified NK cells in preclinical models in vitro and in vivo [26,40,41,42,43,44,45,46,47,48,49,50,51,52,53,54,55]. An important step toward the clinical use of CAR-modified NK cells was the development of a CD19-directed cell product that is based on umbilical cord-derived NK cells transduced with a CD19 CAR construct, is interleukin (IL)-15 expressing, and contains an inducible caspase 9-based suicide gene. This cell product exhibited potent cytotoxicity and long-term persistence in preclinical models [26]. The demonstration of a response in 8 of 11 patients with relapsed or refractory CD19-expressing B cell malignancies treated with these NK cells as part of a phase 1/2 trial, in conjunction with the lack of significant cytokine release syndrome (CRS), immune effector cell-associated neurotoxicity syndrome (ICANS), or GVHD [27] will undoubtedly provide a strong impetus for further exploration of CAR-modified NK cells or NK/T cells.

## 3. The Quest for Target Antigens Suitable for CAR-Modified IEC Therapy

**Figure 2:**
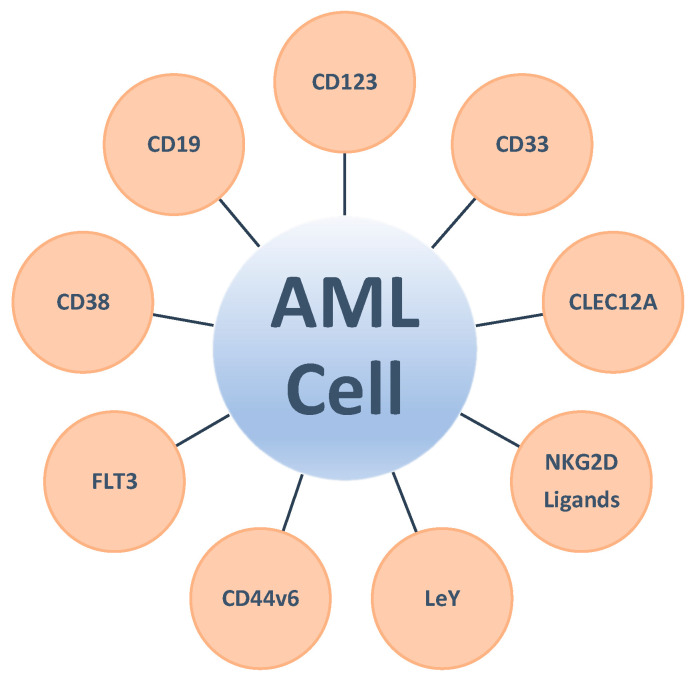
Currently clinically exploited targets for CAR-modified IEC therapy in AML.

The choice of target antigen is critically important for the success of any immunotherapeutic, including CAR-modified IECs. An ideal CAR target is displayed homogenously at high levels in most to all malignant cells including underlying cancer stem and progenitor cells, has minimal or no expression in (vital) normal tissues, is not expressed on engineered IECs to obviate fratricide elimination, is not shed into the circulation, plays a defined role in disease pathogenesis, has a low propensity for antigenic downregulation to alleviate the risk of immune escape, and demonstrates clinical utility [56,57]. In many ways, these requirements are met, for example, with CD19 as a target for the treatment of B cell malignancies, as is reflected by the focus on CD19 in the vast majority of the efforts with CAR-modified IECs in these patients. The situation is fundamentally different in AML, a disease characterized by great immunophenotypic and genetic diversity and sharing of antigens with normal, critically important hematopoietic cells, potentially including normal hematopoietic stem and progenitor cells. This challenge is perhaps best mirrored by the fact that an increasing number of antigens have been explored as targets for immunotherapy in AML because of their (over)expression on AML cells relative to normal cells; their importance for the proliferation, survival, and/or drug resistance of AML cells; their expression on putative AML stem cells; or their expression on cells of the microenvironment that mediate immune responses against leukemic cells over the last several years. Consistent with this, a multitude of targets have been pursued in a first series of clinical trials with CAR-modified IECs in AML (Figure 2). Many additional targets have been proposed based on preclinical studies. Overall, compared to their use in lymphoid malignancies and some other tumors, the clinical experience with CAR-modified IECs in AML is so far very limited. Nevertheless, emerging data from these initial trials highlight not only the uncertainty regarding ideal target antigen selection, but also challenges related to the efficacy and duration of response.

### 3.1. Clinically Exploited Targets for CAR-Modified IEC Therapy in AML

#### 3.1.1. CD123 (IL-3 Receptor α-Chain)

Currently by far the most widely pursued target for CAR-modified IEC therapy for AML is CD123, with a larger number of clinical trials ongoing (e.g., NCT02159495, NCT03114670, NCT03190278, NCT03766126, NCT03796390, NCT04014881, NCT04109482, NCT04230265, NCT04265963, NCT04272125, and NCT04318678). CD123 is widely displayed on blast cells of patients with AML (50% to nearly 100%) and has adverse prognostic significance [58,59]. Like CD33, or any of the other clinically pursued targets, it is not an AML-specific antigen. Rather, within the hematopoietic system, CD123 is displayed on myeloid precursors, monocytes, basophils, plasmacytoid dendritic cells, and a small subpopulation of B cells. It is also found on a broad variety of non-hematopoietic tissues, with high expression on multiple epithelial tissues of the respiratory and gastrointestinal tract. What makes it particularly attractive from a therapeutic perspective is that CD123 is overexpressed on AML stem/progenitor cells relative to normal hematopoietic stem/progenitor cells, which express little or no CD123 [58].

CD123-directed CAR-modified T cells have shown exquisite potency in preclinical AML models [60,61,62,63,64]. Of potential translational interest, the in vitro and in vivo anti-leukemia activity of CD123-directed CAR T cells could be further improved with an azanucleoside such as decitabine [65]. Early data from testing in patients suggest some clinical activity as well, e.g., using a 2nd generation 4-1BB-containing CAR construct expressed in donor-derived T cells, a complete remission with incomplete hematologic recovery (CRi) was obtained in an adult with post-transplant AML relapse [66]. As a second example, among six patients with refractory AML treated with 2nd generation CD28-ζ-EGFRt+ CAR T cells (MB-102) after lymphodepleting chemotherapy with fludarabine/cyclophosphamide, responses were noted in five, with three exhibiting morphological responses [67]. None of the patients exhibited severe CRS or ICANS. Perhaps contrary to some concerns, cytopenias were not dose-limiting in these patients. Another concern with CD123-directed therapies is the expression of this antigen on vascular endothelial cells, which has been invoked as the reason for the development of potentially fatal capillary leak syndrome observed with CD123-directed CAR T cells that led to a clinical hold of that particular cell product. Concerns of toxicity toward normal cells with CD123 CAR-modified T cells prompted efforts to develop [68] and subsequently test an mRNA electroporated (“biodegradable”) CAR construct to shorten the in vivo persistence of engineered T cells. However, all of the five adults with relapsed/refractory AML who were infused with such cells showed disease progression within 28 days of adoptive cell transfer, leading to early termination of this study [69].

#### 3.1.2. CD33

Most efforts to date with antibody-based therapeutics in AML drugs have focused on the myeloid differentiation antigen CD33 [70,71]. That is because CD33 is expressed on at least a subset of AML blasts in almost all cases and possibly leukemia stem cells in some patients [70,71]. The experience has demonstrated that CD33 is a challenging drug target, and several CD33-directed drugs have failed in the clinical testing because they lacked sufficient efficacy [71,72,73]. The relatively low and variable display of CD33 on AML cells—there are on average ~10^4^ CD33 molecules per AML blast, and antigen density varies greater than 2-log-fold across individual patients—may be one reason to account for this difficulty. Still, reduced relapse rates and improved survival, seen in several large randomized trials in at least a subset of patients with newly diagnosed AML when the CD33 antibody–drug conjugate gemtuzumab ozogamicin (GO) is added to intensive chemotherapy, validates CD33 as the first and so far only immunotherapeutic target in AML [74]. Stimulated by both the success of GO as well as its limitations, CD33 has remained of great interest for drug development in AML and a widening range of other malignant and non-malignant diseases, with ongoing efforts with several classes of therapeutics, including newer-generation antibody–drug conjugates, radiolabeled antibodies, bi- and tri-specific antibodies, as well as CAR-modified IECs [72,75].

Preclinical studies demonstrated potent anti-AML efficacy of CD33-directed CAR-modified T cells [62,76,77,78]. Concerns related to the latter compelled the preclinical development of an RNA-modified “biodegradable” CD33-directed CAR T cell product which exhibited potent but transient antileukemic activity in vitro as one conceptual approach to mitigate myelosuppression [77]. Clinical testing of CD33-directed CAR T cells is ongoing (e.g., NCT03971799), so far with limited data reported in the peer-reviewed literature. One such report involves a 41-year-old patient with relapsed/refractory AML who received an autologous 2nd generation cell product without prior lymphodepletion [79]. A total of 1.12 × 10^9^ cells, of which ~38% were transduced with the CAR construct, were infused. An initial reduction in bone marrow blasts was seen within 2 weeks, but the leukemia progressed overtly 9 weeks after the cell infusion. Clinical reports using CD33-directed CAR NK cells are, so far, also limited. In a small trial in which NK-92-MI cells were transduced with a 3rd generation CD33-directed CAR construct, one measurable residual disease (MRD)-positive remission was observed among three treated patients with relapsed/refractory AML [80]. Other efforts to develop NK and cytokine-induced killer cells expressing CD33-targeted CAR constructs are at the preclinical stage [81,82].

#### 3.1.3. C-Type Lectin Domain Family 12, Member A (CLEC12A; C-Type Lectin-Like Molecule-1 (CLL-1))

CLEC12A has raised interest as a therapeutic target not only because this glycoprotein is widely expressed on AML blasts at diagnosis and throughout the treatment course, but also because it is displayed on putative AML stem/progenitor cells [83,84]. In contrast, it is not thought to be expressed on normal hematopoietic stem/progenitor cells, making it an attractive target for CAR-modified IECs. Preclinical models confirmed high anti-AML efficacy of CAR-modified T cells against CLEC12A [85,86,87]. Emerging data suggest that such cells can by highly efficacious and induce MRD-negative remissions in patients with relapsed/refractory AML [88]. Clinical testing of CLEC12A-directed CAR T cells is ongoing, both with cells targeting CLEC12A alone (e.g., NCT04219163) as well as with cell products targeting an additional antigen (Section 3.2.1).

#### 3.1.4. Natural Killer Group 2D (NKG2D) Ligands

NKG2D ligands have been reported in a wide range of malignancies including AML, but they are not widely expressed on normal adult tissue [89]. The application of 1st generation autologous CD3ζ CAR T cells directed against NKG2D ligands exhibited no CRS or ICANS but also no substantial anti-leukemia efficacy in an early phase trial among seven infused patients with relapsed/refractory AML [90]. In a separate trial using another 1st generation CAR construct (CYAD-01), six objective yet short-lived responses were seen among 13 patients with relapsed/refractory AML or myelodysplastic syndrome (MDS) [91]. Very early data with CYAD-02, a next-generation product in which a non-gene editing approach is used to silence MHC class I chain-related protein A and B (MICA and MICB)—NKG2D ligands that are transiently upregulated on activated CAR T cells, indicate safety and tolerability but efficacy data are not yet available [92]. Other efforts targeting NKG2D ligands with CAR T cells are ongoing (e.g., NCT04167696), and a study using CAR-modified allogeneic NK cells is planned [93]. As DNA methylation has been associated with reduced expression of NKG2D ligands on AML cells and, conversely, exposure to azanucleosides can restore surface expression of these ligands [94], co-treatment with a drug such as azacytidine or decitabine may be a rational strategy to increase target density on AML cells.

#### 3.1.5. Lewis Y Antigen (LeY)

The tumor-associated carbohydrate antigen LeY is expressed not only in a wide range of human carcinomas, but also in AML, with preclinical data indicating that CAR T cells directed at LeY can improve survival of animals bearing LeY-expressing malignant hematopoietic cells [95]. In an early phase 1 trial, four adults aged 64–78 years with relapsed AML received autologous T cells expressing a 2nd generation CD28-ζ CAR construct targeting LeY [96]. At the time of cell infusion, three of these patients had cytogenetic evidence of MRD whereas one had active leukemia. CAR T cell persistence for up to 10 months was observed with limited myelosuppression. There were transient responses noted in three patients, including one cytogenetic response, but eventually all patients relapsed between 28 days and 23 months after CAR T cell infusion [96].

#### 3.1.6. CD19

CD19 is occasionally expressed on leukemic cells in patients with AML, e.g., in a subset of patients with core-binding factor leukemias and t(8;21)(q22;q22) (AML1/ETO [RUNX1/RUNX1T1]) translocations. While CD19 is typically neither bright nor uniform in these leukemias, efforts using CD19-directed CAR T cells in CD19-expressing AML are ongoing (e.g., NCT03896854 and NCT04257175).

#### 3.1.7. Other Targets for Single Antigen-Directed CAR-Modified IECs

CD44v6 is the isoform variant 6 of the hyaluronic acid receptor CD44, which is a glycoprotein overexpressed in AML, with increased expression correlating with shorter survival [97]. CD44v6 is thought to be absent on normal hematopoietic stem cells [98], making it an attractive therapeutic target. Value as target for CAR-modified IECs comes from preclinical models showing robust anti-AML activity with 2nd generation suicidal anti-CD44v6 CAR T cells harboring a CD28 costimulatory molecule [99]. Early phase clinical exploration is ongoing (e.g., NCT04097301).

FMS-like tyrosine kinase 3 (FLT3, CD135) is one of the most commonly mutated genes in AML, making it a prime target for small molecule inhibitors (e.g., midostaurin and gilteritinib). As a protein, FLT3 is expressed on AML cells in 70–100% of patients [100]. Because FLT3 display on normal hematopoietic cells is restricted to immature cell subsets, this antigen is of some interest as target for antibody-based therapies in AML. CAR T cells targeting FLT3 have been examined in preclinical models using 2nd and 3rd generation constructs [101,102,103,104]. These cells prolonged survival in mice bearing FLT3-expressing leukemias and had a safe toxicity profile, suggesting this to be a lucrative treatment strategy. Additional preclinical models showed synergy between FLT3-targeted CAR T cells and a FLT3 inhibitor, crenolanib, which increased cell surface expression of FLT3 on AML cells [105]. Clinical investigation examining the efficacy and safety of CAR T cells targeting FLT3 is presently underway (e.g., NCT03904069).

CD38 is widely expressed on AML blasts [106]. While it is also displayed on many normal hematopoietic cells, the absence of CD38 on the most immature normal blood cells lends itself as a possible target for adoptive cell therapies in AML, a notion indicated by preclinical models showing anti-AML efficacy of anti-CD38 CAR T cells in vitro [107]. An early report from a study using CD38-directed CAR T cells in six patients with CD38+ AML relapse following allogeneic HCT showed remissions in four, suggesting clinically meaningful anti-leukemia efficacy [108]. Of potential translational interest, CD38 could be upregulated by all-trans retinoic acid (ATRA), a treatment that augmented the cytotoxic activity of the engineered T cells. The therapeutic value of CD38 targeting with CAR T cells in AML is presently undergoing investigation (e.g., NCT04351022).

### 3.2. Emerging Targets for CAR-Modified IEC Therapy in AML and Emerging Strategies to Enhance Their Antitumor Activity

Beyond the targets already explored in clinical studies, there is no shortage of additional antigens for which preclinical studies have demonstrated that CAR-modified IECs can exert significant cytotoxic activity. Some examples of these efforts include CAR constructs against CD4 [109], CD7 [110], CD25 [111], CD70 [112], CD117 [113], B7-H3 [114], folate receptor β (FRβ) [115,116], glucose-regulated-protein 78 (GRP78) [117], leukocyte immunoglobulin-like receptor-B4 (LILRB4) [118], and mesothelin [119], as well as CAR constructs that entail extracellular portions of NKp44 [120]. It is expected that some of these efforts will be translated into clinical trials over the next several years.

While there are concerns regarding the on-target, off-tumor cell toxicities with CAR-modified IECs in AML (see Section 5), the available clinical data with such therapies also suggest challenges with insufficient anti-leukemia efficacy. Therefore, approaches that enhance the antitumor activity of CAR-modified IECs will likely become of great interest. Many strategies are currently undergoing evaluation. One of these includes the deletion of CD5 in CAR T cells via CRISPR/Cas9, which leads to increased T cell activation, migration, and survival of engineered cells [121]. Others address the pejorative influence of the hostile tumor microenvironment through genetic engineering of IECs to target checkpoint expression on tumor tissue [122,123] or to downregulate T cell expression of checkpoint ligands [124], or through the use of combinatorial approaches with checkpoint inhibitors to augment IEC persistence [125,126].

#### 3.2.1. Value of Targeting Multiple Antigens with CAR-Modified IEC Therapies in AML

The heterogeneity of cell surface antigen expression on AML cells across and within individual patients may favor a polyvalent CAR targeting strategy. The impetus for this combinatorial approach not only comes from the potential to induce more AML-specific cytotoxicity, thereby improving the therapeutic safety of CAR-modified IECs [127], but also from the hope this might increase the therapeutic efficacy [128] and reduce the risk of antigen escape-related disease persistence or relapses. Simultaneously targeting more than one antigen may also allow engineered cells to kill low antigen density tumor cells that otherwise escape CAR-modified IECs [129]. Protein expression profiling of primary AML specimens demonstrated the ubiquitous expression of CD33, CD123, CD244, CLEC12A, and T cell immunoglobulin and mucin domain-containing protein 3 (TIM3; also known as hepatitis A virus cellular receptor 2 (HAVCR2)) in patients with newly diagnosed and relapsed AML irrespective of genetic characteristics [130]. Among these, co-expression analysis revealed the combinations of CD33/TIM3 and CLEC12A/TIM3 were highly expressed on AML cells compared to normal hematopoietic cells [130]. Another complementary antigen to CLEC12A is CD33, given its co-expression in a large proportion of CD33-expressing AMLs [131,132]. Clinical translation of a combinatorial approach is best exemplified by CARs designed to target multiple antigens, or compound CARs (cCARs), with CLEC12A/CD33 co-targeting CAR T cells being an early example. The potential value of cCAR T cells targeting CLEC12A/CD33 in relapsed/refractory AML was first described in a high-risk pediatric patient who achieved an MRD-negative response [133]. An updated analysis of this trial has reported MRD-negative responses in seven of nine treated AML patients 4 weeks after receiving compound CLEC12A/CD33 CAR-T cells [134]. Among the two non-responders, one did not express CLEC12A. In this cohort, reversible CRS and ICANS was reported in high frequency, and all patients exhibited profound myelosuppression meriting HCT support. Of the six patients opting to proceed with allogeneic HCT, only one received myeloablative conditioning prior to transplantation. While the experience is very limited, this trial suggests enhanced therapeutic potential with combinational targeting and hints at the importance of appropriate target selection to extend this therapy to a greater proportion of patients. Preclinical efforts targeting AML with tri-specific CAR constructs are also ongoing [135].

#### 3.2.2. Targeting Neoantigens/Intracellular Antigens with CAR-Modified IEC Therapies in AML

Neoantigen targeting is an emerging therapeutic approach which obviates the off-tumor cell risks feared with nonspecific tumor-associated antigen engagement. Unfortunately, compared to other tumors, such as malignant melanoma or non-small cell lung cancer, AML cells harbor a relatively low number of mutations and, consequently, a lower yield of neoepitopes that could be exploited for therapeutic targeting. Nevertheless, driver mutations in isocitrate dehydrogenase 1 or 2 (IDH1, IDH2), nucleophosmin (NPM1), FLT3, as well as splice variants in other genes (e.g., CD44) exhibit neoepitopes potentially suitable as tumor-specific antigens for the purpose of CAR-modified IEC therapy in AML [136]. While CAR IECs require the expression of surface antigens to exert cytotoxicity in an HLA-independent manner, TCR-mimic CARs are being developed to engage intracellularly processed antigens in an HLA-restricted manner [137]. Preclinical proof-of-principle studies with TCR-mimic CAR constructs targeting WT1 have been reported [138]. This approach was most recently extended in a preclinical model utilizing a TCR-mimic CAR construct targeting HLA-A2-NPM1c-expressing AML in mice [139]. Ex vivo and in vivo cytotoxicity was restricted towards NPM1c^+^HLA-A2^+^ leukemia cells but spared NPM1c negative tumor cells, which may translate into a more favorable toxicity profile and abrogate resistance. Collectively, these approaches may afford a tumor specific approach to addressing AML without the unintended adverse effects of off-tumor toxicities.

## 4. The Immunosuppressive Tumor Microenvironment as an Impediment to the Therapeutic Efficacy of CAR-Modified IECs

The tumor microenvironment originates from, and is predominantly driven by, the cancer cells themselves. It is dominated by tumor cell-induced recruitment of dysfunctional IECs and interactions with these cells via complex interplay with tumor-initiated signals [140]. The importance of the microenvironment for the promotion of tumor growth and attenuated response to anti-cancer therapy is well recognized in many human malignancies and is of relevance for adoptive cell therapy in AML as well, with emerging evidence suggesting the microenvironment may impair the anti-leukemic efficacy of CAR-modified IECs in AML. This conclusion, and information related to possible mechanisms underlying this immune resistance, is currently largely based on extrapolations from the use of CAR T cells in other tumors [141], as data pertaining to AML specifically are still sparse. While many details await elucidation, it has become clear that the tumor microenvironment in AML is complex and that strategies to overcome resistance to CAR-modified IECs due to effects from this environment will require a pleotropic approach to combat dysfunctional immune cells, an unfavorable cytokine milieu, immune dampening metabolic dyscrasias, and upregulation of checkpoint ligands imposed by this disease.

### 4.1. Cellular Components of the Immune Suppressive AML Microenvironment: AML Blasts and Other Immunosuppressive Cells

The leukemic myeloblasts are the makers of the tumor microenvironment in AML. As a result of a myriad of direct and indirect effects (see also Section 4.2 and Section 4.3), there is a robust expansion of regulatory T cells (Tregs), which impair the proliferation of cytolytic T cells and, in conjunction with recruited myeloid-derived suppressor cells (MDSCs), dendritic cells, and polarized inhibitory macrophages [142], dampen T cell activity [143,144,145]. Dysfunctional NK cells with downregulated cell surface expression of activating natural cytotoxicity receptors or expression of inhibitory killer immunoglobulin-like receptors (KIRs) further contribute to the ability of AML cells to evade immune surveillance [146]. Many of these cell types have been shown to curtail the activity of CAR-modified IECs in preclinical models. For example, expanded MDSCs, which impair T cell activity through the production of reactive oxygen species and recruitment of Tregs [147], suppress CAR T cell efficacy in solid tumor models [148,149]. Conversely, depletion of Tregs in a murine AML model of adoptive cell transferred T cells improved cytotoxic T cell expansion [150]. Clinical relevance of such cellular interactions is suggested by the observed relationship between tumor-associated macrophages and CAR T cell activity in 10 patients with chemotherapy-refractory diffuse large B cell lymphomas treated with CD19-directed CAR T cells in the setting of a phase 1 dose-escalation study, in which remission status after CAR T cell therapy was negatively influenced by an increased infiltration with tumor-associated macrophages, as determined in tumor samples collected via core needle biopsies [151].

### 4.2. Alterations of the Cytokine Milieu

The cytokine and chemokine milieu may play an important role in the proliferative and effector function of CAR-modified IECs [152]. While AML blasts can prompt the secretion of stimulatory cytokines such as tumor necrosis factor (TNF)-α, IL-1β, and IL-6 by monocytes, upregulation of inducible T cell costimulator ligand (ICOSLG) on AML blasts can provide co-stimulation through ICOS for the conversion and expansion of Tregs sustaining high Foxp3 and CD25 expression and a suppressive cell function with secretion of inhibitory cytokines, including transforming growth factor (TGF)-β and IL-10 [153]. The latter not only promote AML cell growth [153], but also reduce the activity of CAR T cells [154,155]. Additional downregulation of various transcription factors and stimulatory cytokine receptors (e.g., IL-2 and IL-15) may further exacerbate the immunosuppressive microenvironment in AML [146,156].

Chemokines and their respective receptors have been implicated in the trafficking of IECs, including CAR-modified IECs, to tumor sites. For instance, the co-expression of the chemokine receptor C-C chemokine receptor type 4 (CCR4) with a CAR construct increased the accumulation of cytotoxic T cells in a Hodgkin lymphoma model [157]. Moreover, expression of the chemokine ligand CCL19 with IL-7 enhanced the infiltration of CAR T cells into solid tumors in mice [158]. 

Together, the disproportionate expansion of immunosuppressive Tregs may blunt a potent IEC response. Clinically, eradication of Tregs, downregulation of indolamine 2,3-dioxygenase (IDO; see Section 4.3), and improvement of the cytokine milieu are attempted with the use of lymphodepleting chemotherapy prior to CAR-modified IEC infusion, a strategy that may lead to improved treatment outcomes as indicated by studies in non-Hodgkin lymphoma [159]. Additional strategies to optimize IEC efficacy may include the provision of co-stimulation of engineered cells via 4-1BB signaling to overcome Treg-induced immunosuppression [160], perhaps through use of 4-1BB signaling components, use of CAR-modified cells with transgenic cytokine co-expression [161], and complementary checkpoint inhibition (see Section 4.4). 

### 4.3. Metabolic Dysregulation

Metabolic dysregulations of tryptophan, glutamine, arginine, and adenosine provide a mechanism of promoting tumor cell growth and immune escape in AML [145,162]. Tryptophan metabolism is highly regulated, and its physiologic depletion inhibits T cell proliferation. The AML microenvironment exploits this through the expression of IDO, an enzyme which catalyzes the degradation of tryptophan. IDO expression is prevalent in AML blasts and the pathologic bone marrow niche with a higher frequency of circulating Tregs [163]. Pharmacologically blocking IDO, e.g., via 1-methyl tryptophan [164], can enhance the activity of conventional AML therapeutics [165]. Such inhibitors may also be of interest with CAR-modified IEC therapy in AML. This notion is based on preclinical data showing impaired proliferation, cytotoxicity, and cytokine secretion of CAR T cells against IDO-expressing tumors. While costimulatory molecule expression on CAR T cells did not overcome the immunosuppressive effects of IDO, it could be overcome with 1-methyl tryptophan or the use of lymphodepleting chemotherapy, which was found to downregulate IDO expression [166].

The microenvironment in AML is also rich in glutamine, which inhibits T cells, leading to cellular exhaustion and, ultimately, enhanced leukemic cell survival [167]. Inhibition of glutamine uptake may afford a therapeutic approach to overcome the immune dysfunction imposed through this mechanism [168]. AML blasts endorse a microenvironment with low levels of arginine through accentuated catabolism by arginase II. The lack of arginine results in impaired secretion of stimulatory cytokines such as interferon (IFN)-γ and increased checkpoint inhibitor ligand expression, which attenuate IEC response and proliferation [169]. Repleting arginine improves CAR T cell activity against AML in preclinical studies [170]. Last, excess levels of adenosine impair T cell activity in the AML microenvironment and are facilitated through the ectonucleotidase expression of CD38, CD39, and CD73 on myeloid blasts which metabolize adenosine triphosphate (ATP) [171,172]. Inhibition of adenosine signaling through adenosine 2A receptor blockade may facilitate enhanced CAR T cell activity, and monoclonal antibodies to restore adenosine metabolism may mitigate adenosine based immune evasion [171,173].

### 4.4. Immune Evasion through Checkpoint Ligands

AML blasts have demonstrated a proclivity for immune evasion through the downregulation of MHC molecules while continuously expressing immune checkpoint ligands such as programed death-ligand 1 (PD-L1), B7-H3, lymphocyte-activation gene 3 (LAG3), TIM3, galectin 9 (Gal-9), and cytotoxic T cell lymphocyte associated protein 4 (CTLA-4) [174,175,176,177]. The observation that upregulation of these ligands can lead to T cell exhaustion [145,178] provides the impetus for the use of checkpoint inhibitors and reverse targeting to enhance the antitumor efficacy of CAR-modified IECs—a concept supported by emerging clinical data with CAR T cells in patients with B cell malignancies [125,126]. The upregulation of programmed death-1 (PD-1) on CAR T cells and PD-L1 on tumor cells has been described subsequent to infusion of CD19-directed CAR T cells and may underlie treatment failure in some patients [179,180]. This mechanism of immune escape has propelled interest the combining CAR T cells with checkpoint inhibitors in B cell lymphomas [125,126]. Conceivably, this approach could also enhance the efficacy of such cells in AML, and novel strategies exploiting the expression of checkpoint ligand-directed CAR constructs [123,181,182], gene editing techniques to remove checkpoint expression on CAR-modified IECs [124,183], and engineering cells to also secrete checkpoint blocking scFvs [122] are of interest in various malignancies. As one example of such interests, studies in mice showed safety and anti-AML efficacy of a dual-targeted CAR against CD13 and TIM3 [123], a known culprit of T cell exhaustion that, as mentioned above, is expressed on AML cells including putative AML stem cells [184,185]. An engineered bispecific CAR targeting these two molecules was able to eradicate leukemia while sparing the healthy bone marrow compartment, highlighting the potential value of exploiting targets in the tumor microenvironment when engineering novel adoptive cell transfer approaches for AML. However, careful testing of checkpoint targeting and inhibition will be necessary not only due to concerns of expected toxicities, but also because, unlike in many other human cancers, the value of immune checkpoint inhibition in AML is currently unclear, with some benefit suggested in smaller, uncontrolled studies [186,187] but early data from a randomized trial indicating no outcome improvement when used with an azanucleoside [188].

### 4.5. Antigen Escape after CAR-Modified IEC Therapy

Antigen escape, or loss of antigen density, has been recognized as a mechanism underlying tumor relapse in patients treated with CAR T cells directed at a variety of antigens, including CD19 (B cell malignancies), B cell maturation antigen (BCMA (multiple myeloma)), and epidermal growth factor receptor variant III (EGFRvIII (glioblastoma multiforme)) [160,189,190]. This cause of treatment failure is particularly well established in pediatric and adult patients treated with CD19-directed CAR T cells for B-ALL, in which reported frequencies of CD19-negative escape after CAR T cell therapy range between 7% and 25% [17,189,191,192,193,194,195]. This phenomenon is likely correlated with the sustained antigenic pressure imposed by persisting CAR T cells, accounting for the higher likelihood of CD19 loss in patients treated with 4-1BB-containing CARs as compared to those treated with CD28-containing CARs [195]. Strategies to overcoming antigen escape are currently being developed in B cell malignancies, e.g., with the use of polyvalent targeting CAR-modified IECs.

A novel antigen escape mechanism reported in ALL entails class switching to an acquired myeloid phenotypic leukemia, as seen in two patients with *MLL*-rearranged ALL treated with anti-CD19-directed CAR T cell therapy [192]; in principle, similar class switching phenomena could occur in such leukemias when treated with potent CAR-modified IECs directed at myeloid antigens. While antigen loss has not yet been widely reported with CAR-modified IECs in AML, the concern for loss of the target antigen, effectively nullifying the significance of persistence of engineered IECs, also applies to this malignancy. Depending on their therapeutic intent, this mechanism of immune escape may lead to the need for a consolidative HCT in this population.

## 5. Toxicity Considerations for CAR-Modified Immune Effector Cells in AML and Possible Mitigation Strategies

The characteristics and severity of side effects associated with CD19- and BCMA-directed CAR T cells including CRS, ICANS, infusion reactions, tumor lysis syndrome, cytopenias, hypogammaglobulinemia, and cardiac events are well established, and toxicity management algorithms have been developed [196,197,198,199,200,201,202,203]. Some of these adverse events are viewed as “on-target, off-tumor cell” toxicities given the expression of the target antigen on non-diseased tissue (e.g., expression of CD19 on normal B cells, leading to hypogammaglobulinemia with CD19-directed CARs). Others (e.g., CRS) are largely a manifestation of the activation of the engineered IECs and can occur whether cell engagement is via target antigen displayed on diseased or non-diseased tissue.

While cross-trial comparisons and immediate translation of available data related to risk for CRS and ICANS are nearly impossible between individual CAR-modified IEC products given differences in the cellular manufacturing approach, cell dose, disease characteristics, lymphodepleting chemotherapy regimens, trial design, toxicity grading, and the patients treated, some risk factors for CAR T cell-mediated toxicity in CD19-expressing B cell malignancies have been identified. In a large single institutional study reporting on patients with B cell malignancies receiving autologous 2^nd^ generation 4-1BB-containing CD19-directed CAR T cells, risk factors for the development of CRS included the CAR T cell dose, use of fludarabine-containing lymphodepleting conditioning therapy, bone marrow involvement of disease (particularly with B-ALL), and the degree of CAR T cell expansion following cellular infusion [204]. Data with 1^st^ generation CD19-directed CAR T cells exhibiting a very low risk of high-grade CRS and ICANS, but also inferior outcomes when compared to later-generation CAR T cell products, suggest an important role of cellular engineering and relationship between IEC potency and toxicity. Furthermore, the immunostimulatory potential of the costimulatory molecule incorporated in the CAR construct and the cellular composition (e.g., ratio of CD4:CD8 CAR-modified T cells) may account for the disparate CRS and ICANS rates between reported 2nd generation autologous CD19-directed CAR T cell products for aggressive B cell lymphomas [18,205,206,207]. Some of these differences have gained relevance for treatment decision-making: for example, the lower frequency of CRS and ICANS with 2nd generation 4-1BB-containing CAR constructs as compared to those harboring a CD28 costimulatory domain may influence cell product selection for patients considered for CD19-directed CAR T cell therapy. 

It is likely the specifics of the cell type used in the engineered IEC product has a major impact on the toxicity profile. This notion is supported by data from a recent phase 1 clinical trial deploying IL-15-secreting “armored” CD19 CAR NK cells in 11 patients with relapsed/refractory B cell malignancies, in which a high rate of responses without CRS or ICANS were noted [204]. While preliminary, this study hints at the possibility of a safer delivery of CAR modified IECs without compromising outcomes in B cell malignancies—an observation that could conceivably extend to AML. Nevertheless, it is important to note that extrapolating toxicity risks from the experience of IEC experiences in other malignancies, and even within AML, may have to consider discrepancies not only in the cellular product and patient population as previously stated, but also the approach to the management of toxicities. The latter is largely contingent upon the provider’s and institutional IEC experience, despite existing (yet evolving) guidelines in this nascent field. 

It is currently unclear to what degree toxicities with CAR-modified IECs in AML will parallel those seen in other malignancies, both with regards to the nature and severity of the toxicity. Contrasting toxicity data from CD19-directed CAR T cell trials with maturing data from several trials using CAR T cells in multiple myeloma may inform to what degrees toxicity profiles differ with the change of the target antigen—information that may help anticipate toxicity profiles with CAR-modified IECs in AML. Given that only a small number of AML patients treated with CAR T cells in AML have been reported to date, actual clinical data are sparse. However, the published experience with CD33 and CD123 CAR T cells in relapsed/refractory AML indicates a high frequency of high-grade CRS with the use of 2nd generation CAR T cells [69,79].

Perhaps even more challenging, with currently pursued targets, unintended myeloablation from “on-target, off-tumor cell” engagement of normal hematopoietic stem and progenitor cells through targeting of overlapping myeloid antigens may lead to potentially fatal consequences for those unable to receive rescue hematopoietic stem and progenitor cell products (e.g., allogeneic HCT). The experience with CD33-targeted therapeutics is a paradigm for the challenges when targeting antigens that are not AML specific but also displayed by normal cells. CD33 is expressed on multipotent myeloid precursor cells, unipotent myeloid colony-forming cells, and maturing myeloid cells and monocytes [70,71,208]. Therefore, unwanted CD33-specific “on target” toxicities toward these cells must be expected with the use of potent CD33-targeted therapies, manifesting primarily as prolonged myelosuppression and associated risks of infections and/or bleeding. In addition, hepatic toxicities may occur as a consequence of the targeting of CD33-expressing cells in the liver (e.g., Kupffer cells and sinusoidal endothelial cells) [74,209]. This expression profile leaves a narrow therapeutic window for CD33-targeted drugs. This is well illustrated with the use of GO, where sinusoidal obstruction syndrome/veno-occlusive disease (SOS/VOD) is a feared toxicity and increased deaths related to myelosuppression can offset any anti-leukemia efficacy benefits [210,211]. This is also exemplified by the fate of vadastuximab talirine (SGN-CD33A) [73]. As a second generation antibody-drug conjugate, it integrated improvements in conjugation methods, linker technology, and potency of the cytotoxic moiety over GO [212]. While vadastuximab talirine demonstrated single-agent activity in relapsed/refractory AML, concerns over toxicity to the liver impacted clinical testing. Ultimately, the development of this drug was discontinued after a randomized phase 3 study testing the addition of vadastuximab talirine to azacitidine or decitabine in patients with previously untreated intermediate- or adverse-risk AML was terminated prematurely because of a higher rate of deaths, including fatal infections, in the vadastuximab talirine-containing arm [73].

Ideally, future research would identify a target antigen with very limited (or absent) expression on normal maturing and mature hematopoietic cells. For other targets, therapeutic risks may impose the need for mitigation strategies to allow reasonably safe use of CAR-modified IECs in AML. Improvements in supporting patients with profound, prolonged myelosuppression and ability to rescue patients with allogeneic or (tumor-free/purged) autologous hematopoietic stem and progenitor cells may be important strategies to render use of highly potent CAR-modified IECs safer in these situations. In principle, strategies that curtail the efficacy of CAR-modified IECs could be employed to attenuate myeloablative risks from protracted off-tumor targeting and increase their safety. Such approaches may entail the selection of a CAR construct with more limited in vivo persistence (e.g., using a CD28 costimulatory domain rather than a 4-1BB costimulatory domain [213]). Other means to abrogate excessive myeloablative T cell activity may include the use of a suicide gene such as iCaspase9 [214]; inserting a co-expressed truncated surface antigen such as EGFR, CD19, or CD20 to facilitate engineered IEC clearance with an approved monoclonal antibody [215]; or through nonviral engineering utilizing degradable mRNA which may result in transient cellular persistence [69]. Last, nonspecific pharmacological strategies including anti-thymocyte globulin, alemtuzumab, and corticosteroids could be potentially used to attenuate accentuated CAR-modified IEC toxicity due to their lympholytic effects [198,216].

Of course, deliberately curtailing the persistence of CAR-modified IECs may increase the risk of inadequate therapeutic response and facilitate leukemia cell survival. An alternative strategy to widen the therapeutic window for the clinical use of these cells while preserving efficacy is to genetically engineer normal hematopoietic stem and progenitor cells such that they no longer express significant amounts of the target antigen to mitigate toxicity to normal cells. This strategy will be reserved for CAR-modified IECs targeting antigens that are expressed on, but are non-essential for, normal hematopoietic cells. Nevertheless, this could provide a clinically exploitable strategy in AML, and CD33 serves as a paradigm for this approach. Specifically, a potential breakthrough in bringing the concept of using engineered normal hematopoietic cells to mitigate toxicities of targeted therapy has been the observation that normal hematopoietic stem and progenitor cell populations can be engineered to lack CD33 (e.g., via CRISPR/Cas9) seemingly without resulting impairment of engraftment potential or other functionality and resist potent CD33-directed therapeutics, including CAR-modified IECs [217,218,219]. This might enable the use of engineered hematopoietic stem cell products in patients considered for CD33-directed adoptive cell therapies. This therapeutic strategy will soon be tested in the clinic.

## 6. Different CAR Constructs for Different Clinical Scenarios?

At the present time, clinical investigations exploring the safety and efficacy of genetically modified IECs in AML are focused on patients with relapsed or refractory disease. In this setting, the use of adoptive cell therapies is often promptly followed by an allogeneic HCT [67,134]. However, as the field advances, explicitly defining the therapeutic intent of adoptive cell transfer as either a bridge or a destination therapy will be of importance when selecting the “ideal” cellular CAR construct and respective target(s). Given the sparse and vastly heterogeneous data examining CAR-modified IECs in most disease states, it is difficult to establish categorical edicts regarding the single best approach in AML. Nevertheless, it is rational to assume that the most appropriate cellular product selection in the management of AML must take into account the therapeutic intent with its use given the clear implications of cellular approach on maximal therapeutic potency, cellular persistence, on-target toxicity, and reliable immunosurveillance. For instance, if CAR-modified IEC therapy is to be utilized as a bridge to allogeneic HCT, a clinically tolerable cellular construct “simply” capable of inducing maximal tumor eradication to achieve MRD-negativity would be of paramount significance. Longer-term toxicity to normal hematopoietic cells would obviously be less of a problem, although adverse effects toward other normal tissues would still need to be an important consideration. On the other hand, if CAR-modified IECs are used as a destination therapy, either by virtue of supplanting the need for an allogeneic HCT or among transplant-ineligible patients, the CAR construct most capable of potent cytolytic activity for effective tumor eradication while maintaining long term persistence in order to mitigate relapse risk may be of particular relevance. This intent to exert favorable long-term survival through the sole use of CAR-modified IECs may require a CAR construct capable of overcoming cellular exhaustion, encompassing metabolic endurance to maintain cellular persistence, and harbor leukemia-specific polyvalent targets to counter the potential for antigen-escape leukemic relapses from sustained antigenic pressure yet minimize toxicity to normal (hematopoietic) tissues. While present cellular immunotherapy data do not allow for head-to-head comparisons between cellular products or types of CAR constructs, 2nd generation CD28 and 4-1BB CAR constructs have been the basis of a robust clinical experience in the management of CD19-expressing B cell malignancies. Based on this experience, cellular persistence may be better achieved through the use of 4-1BB-containing CAR constructs over those containing CD28 costimulatory domains albeit with a higher risk of antigen-escape, thereby invoking the need for multi-target approaches to maximize clinically durable benefit in AML. With all these considerations, a single best CAR construct (“one fits all”) may not exist.

## 7. Conclusions

Adoptive cell transfer with CAR-modified IECs has caused much excitement in the ongoing attempts to use targeted immune-based therapies to improve outcomes in AML—not only for patients with relapsed or refractory disease (the focal point of current investigations), but also for earlier uses, e.g., after seemingly successful post-remission therapy. In the latter settings, CAR-modified IECs may serve as a potential bridge to allogeneic HCT. This may not only ensure maximal tumor eradication, thereby improving survival, but potentially obviate the need for toxic myeloablation prior to allografting. While untested, this approach could allow for safer delivery of pure hematopoietic progenitor stem cells deplete of ancillary T cells and mitigate non-relapse morbidity/mortality from GVHD.

Although CAR-modified IECs have been highly efficacious in B cell malignancies, immediate translation of this success to AML is difficult due to several factors discussed in this review. First, the lack of an “expendable” AML-specific target to mitigate potentially lethal on-target, off tumor toxicity poses a formidable, hitherto unresolved challenge. Potential strategies to address this limitation may include the use of potent yet biodegradable CAR constructs, persistence-limiting costimulatory molecules within CAR constructs, multi-targeted leukemia-specific CAR constructs to increase the leukemia specificity of the engineered cells, and/or through the design of genetically engineered constructs with safety switches to limit CAR-modified IEC persistence to curtail off-tumor toxicity. The second limitation lies in the inherently contentious tumor microenvironment in AML with a high proportion of immunosuppressive cells, metabolic dysfunction, upregulation of various checkpoint ligands, and proclivity for antigen escape impairing AML directed cytotoxicity. Thus, regardless of the cellular product and construct, combinatorial strategies considering the hostile tumor microenvironment may be required to maximize the clinical efficacy of CAR-modified IECs. An additional logistical challenge with regards to the use of adoptive cell transfer must take into account the rapidly proliferative kinetics of AML. Therefore, cellular products which allow for an “off-the-shelf” allogeneic gene-modified IEC may be more favorable, stipulating reliable mitigation of GVHD risks to the patient. As discussed, this may be accomplished through gene editing of T cells to remove the receptors posing GVHD risk or through the potential use of CAR-modified NK cells which dispel potent tumor directed cytolytic activity without the respective risk for GVHD.

At the present time, it is unclear if gene-modified IECs will serve as destination therapy and supplant the need for a conventionally “curative” allogeneic HCT in the management of AML. Nevertheless, the successful displacement of this conventional approach may require not only a potent engineered IEC capable of profound leukemic eradication, but one that is capable of exhibiting cellular persistence while overcoming the various pleotropic mechanisms of leukemia escape. The proverbial race to CAR-modified IEC approval in AML will require a creative and comprehensive approach to address the complex interplay between the disease, its tumor microenvironment, and subtle but important nuances of delivering cellular immunotherapy.
